# Supplementary material for: Robust derivation of transplantable dopamine neurons from human pluripotent stem cells by timed retinoic acid delivery
Source: Nat Commun. 2022 Jun 1;13:3046. doi: 10.1038/s41467-022-30777-8 (PMC9160024; doi:10.1038/s41467-022-30777-8)
Supplement: Supplementary file 1 — Supplementary Information [file 41467_2022_30777_MOESM1_ESM.docx]

**Supplementary Information**

Robust derivation of transplantable dopamine neurons from human pluripotent stem cells by timed retinoic acid delivery

Zhanna Alekseenko, José M. Dias, Andrew F. Adler, Mariya Kozhevnikova, Josina Anna van Lunteren, Sara Nolbrant, Ashwini Jeggari, Svitlana Vasylovska, Takashi Yoshitake, Jan Kehr, Marie Carlén, Andrey Alexeyenko, Malin Parmar, Johan Ericson

**
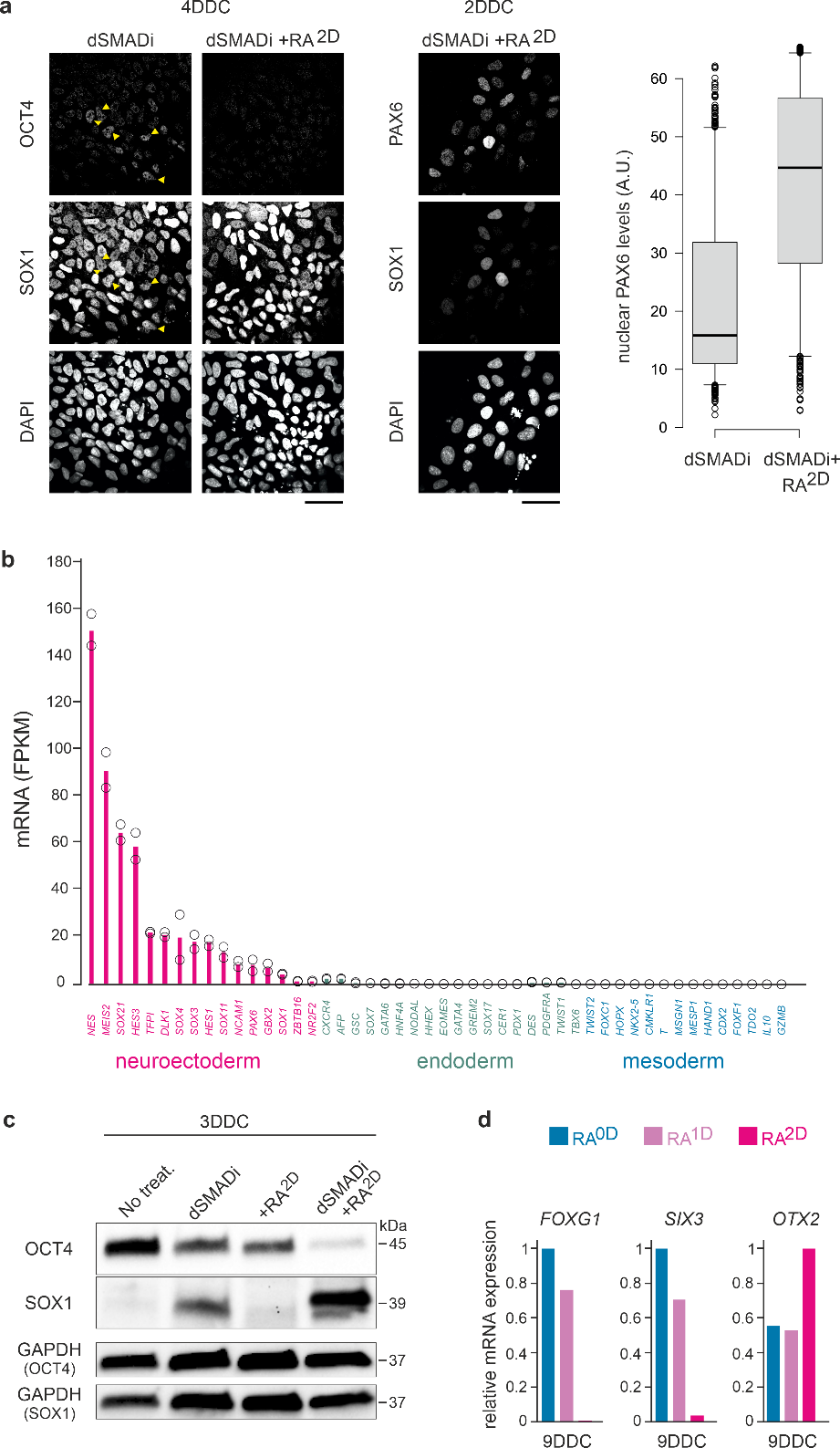
**

**Supplementary Fig. 1: Combination of RA and dSMADi results in a rapid induction of neuroectoderm from human PSCs. a** Immunocytochemistry for the pluripotency marker OCT4 and the neuroectodermal markers SOX1 and PAX6 on 4-day and 2-day cultures differentiated in dSMADi or in dSMADi+RA^2D^ conditions. Yellow arrows (left side panel) indicate OCT4^+^/SOX1^+^ cells. Boxplot of PAX6 nuclear level (right) in 3 DDC cultures differentiated in dSMADi (n=792 cells) or dSMADi+RA^2D^ (n=761 cells). Data presented as boxplot from 3 independent experiments, the band indicates the median, the box indicates quartile 1 to quartile 3, and whiskers extend to 5th and 95th percentile. **b** RNA-seq expression analysis from 2 independent experiments of genes associated with neuroectodermal, endodermal, and mesodermal lineages in 2 DDC cultures differentiated in dSMADi+RA^2D^. **c** Representative western blot for OCT4 and SOX1 of 3 DDC cultures differentiated in indicated conditions. **d** Q-PCR expression analysis for the genes identifying forebrain (*FOXG1*, *SIX3*), forebrain, and midbrain (*OTX2*) regions in 9 DDC cultures differentiated in dSMADi and treated with indicated RA-pulse. DDC, days in differentiation conditions. Scale bars, 50µm.

**
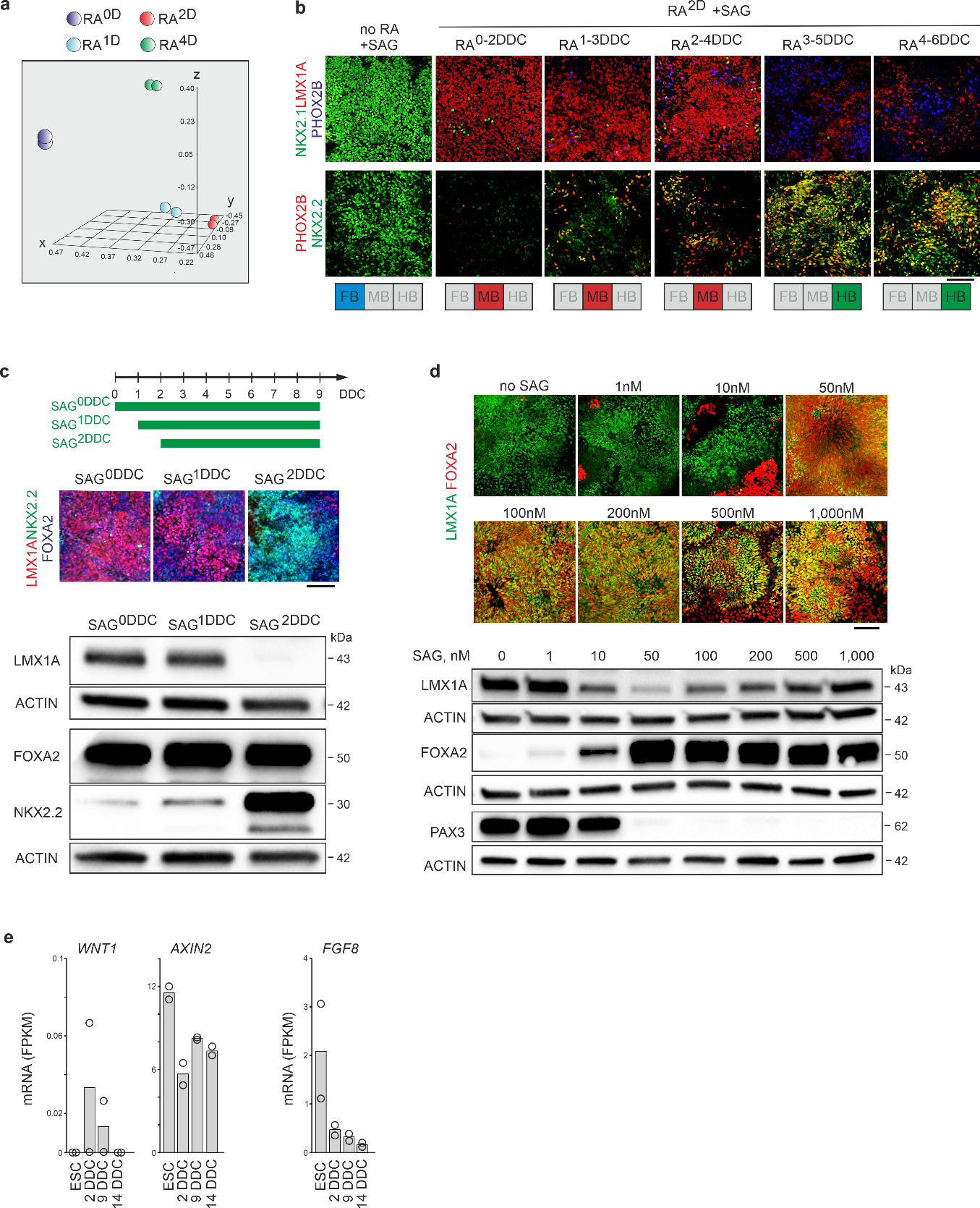
Supplementary Fig. 2: Effect of RA and SAG application time and SAG concentration on NSC specification. a** Principal component analysis plot of 9 DDC cultures differentiated in dSMADi+SAG and pulsed with RA from 0 DDC for 0 (RA^0D^), 1 (RA^1D^), 2 (RA^2D^), or 4 (RA^4D^) days. **b** Immunocytochemistry for NKX2.1, LMX1A, and PHOX2B, or NKX2.2 and PHOX2B in 9 DDC cultures. Cells were differentiated in dSMADi+SAG only (no RA+SAG) or together with a 2-day RA pulse (RA^2D^+SAG) applied at different times during differentiation (0-2, 1-3, 2-4, 3-5, or 4-6 DDC). **c** Schematic of hESC differentiation (in dSMADi-condition) with timeline of SAG addition to cultures (top). Immunofluorescence for LMX1A, NKX2.2, and FOXA2 (middle) and representative western blot for LMX1A, NKX2.2, and FOXA2 (bottom) in 9 DDC cultures treated with SAG from day 0, 1, or 2. **d** Immunofluorescence for LMX1A and FOXA2 (top) and representative western blot for LMX1A, FOXA2, and PAX3 (bottom) in 9 DDC cultures differentiated in dSMADi and treated from day 0 with different SAG concentrations. **e** RNA-seq expression analysis of *WNT1, AXIN2,* and *FGF8* in ESCs and in 2, 9 and 14 DDC cultures differentiated in dSMADi+RA^2D^ in 2 independent experiments.

Scale bars, 100µm

**
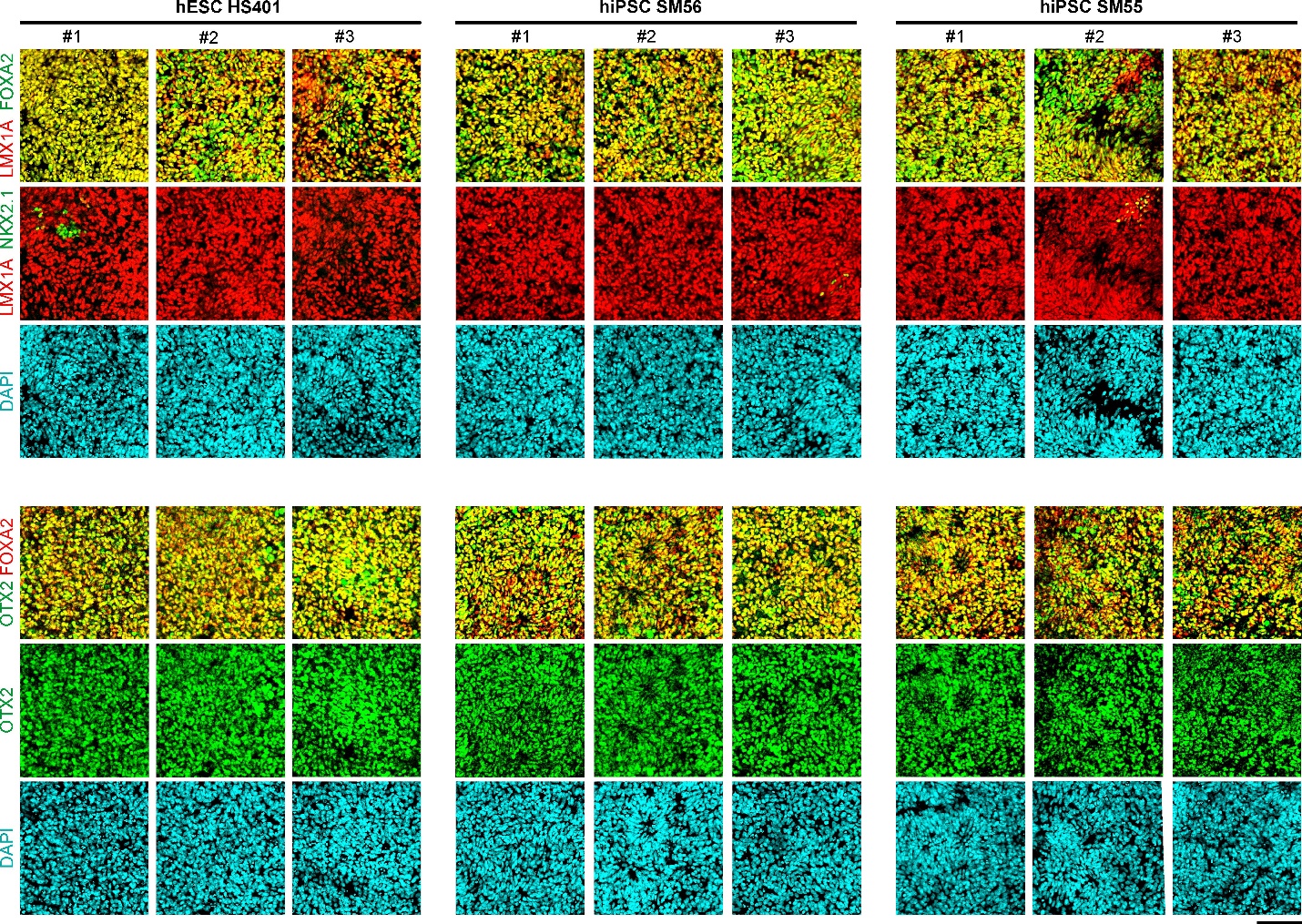
**

**Supplementary Fig. 3: Patterning response to RA^2D^+SAG treatment across additional** **cell lines.** Analysis of LMX1A, FOXA2, NKX2.1, and of OTX2, FOXA2 expression at 14 DDC in three biological independent cultures (#1-#3) differentiated in dSMADi+RA^2D^+SAG using an additional ESC line HS401, and the hiPSC-lines SM56 and SM55. Scale bar, 100 µm

**
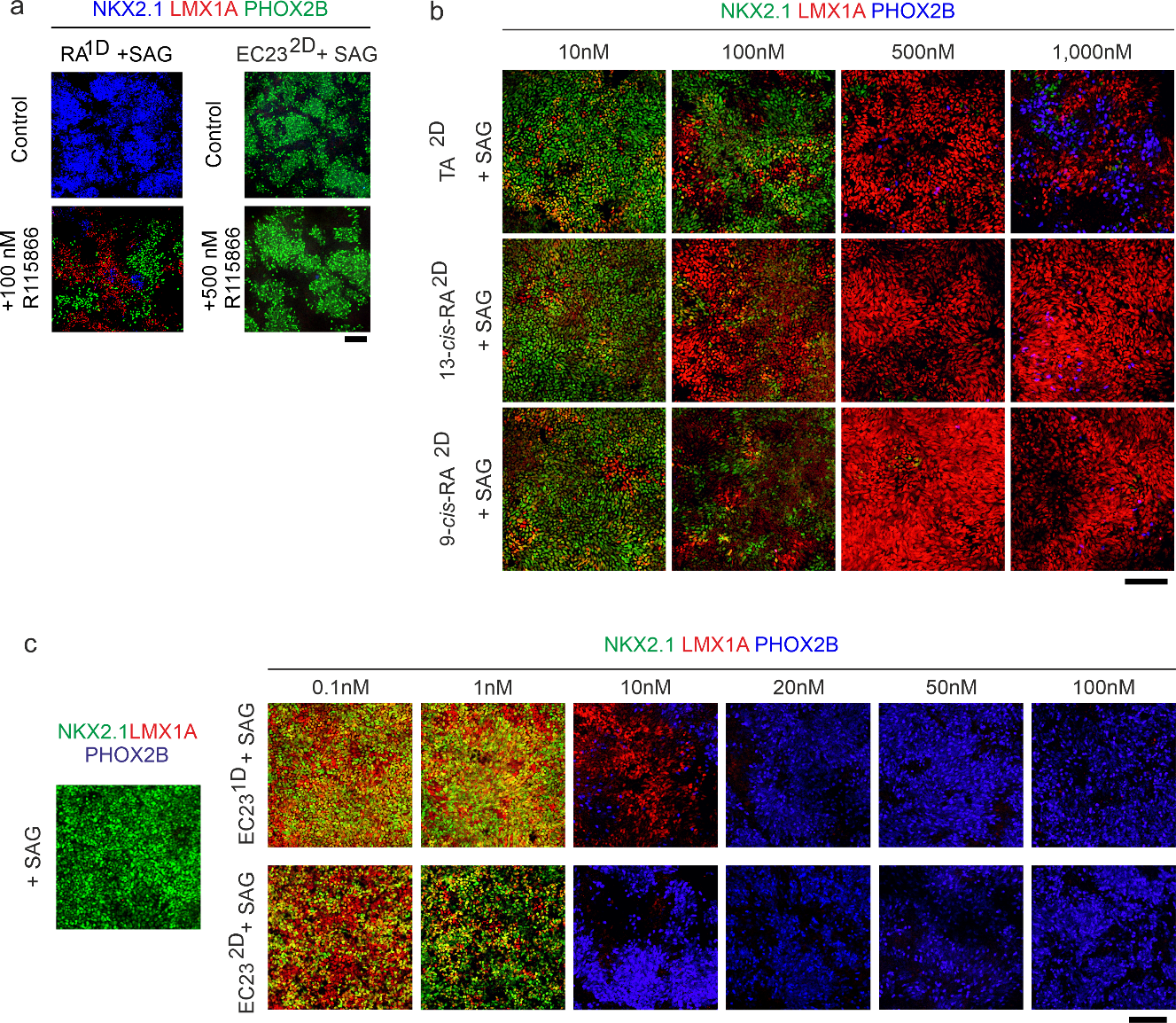
**

**Supplementary Fig. 4: Effect of RA analogues on NSC specification. a** Analysis of NKX2.1, LMX1A, and PHOX2B expression in 9 DDC cultures differentiated in dSMADi+SAG and pulsed with RA for 1day or EC23 for 2 days in the presence or absence of the inhibitor R115866. **b** Analysis of NKX2.1, LMX1A, and PHOX2B expression in 9 DDC cultures differentiated in dSMADi+SAG and pulsed with the all*-trans-*RA-analogues tazarotenic (TA), 13*-cis-*RA, or 9*-cis-*RA for 2 days at different concentrations (10, 100, 500, and 1,000 nM). **c** Analysis of NKX2.1, LMX1A, and PHOX2B expression in 9 DDC cultures differentiated in dSMADi+SAG and pulsed with EC23 at the indicated concentrations for 1(EC23^1D^) or 2 (EC23^2D^) days. Scale bars, 100 µm

**
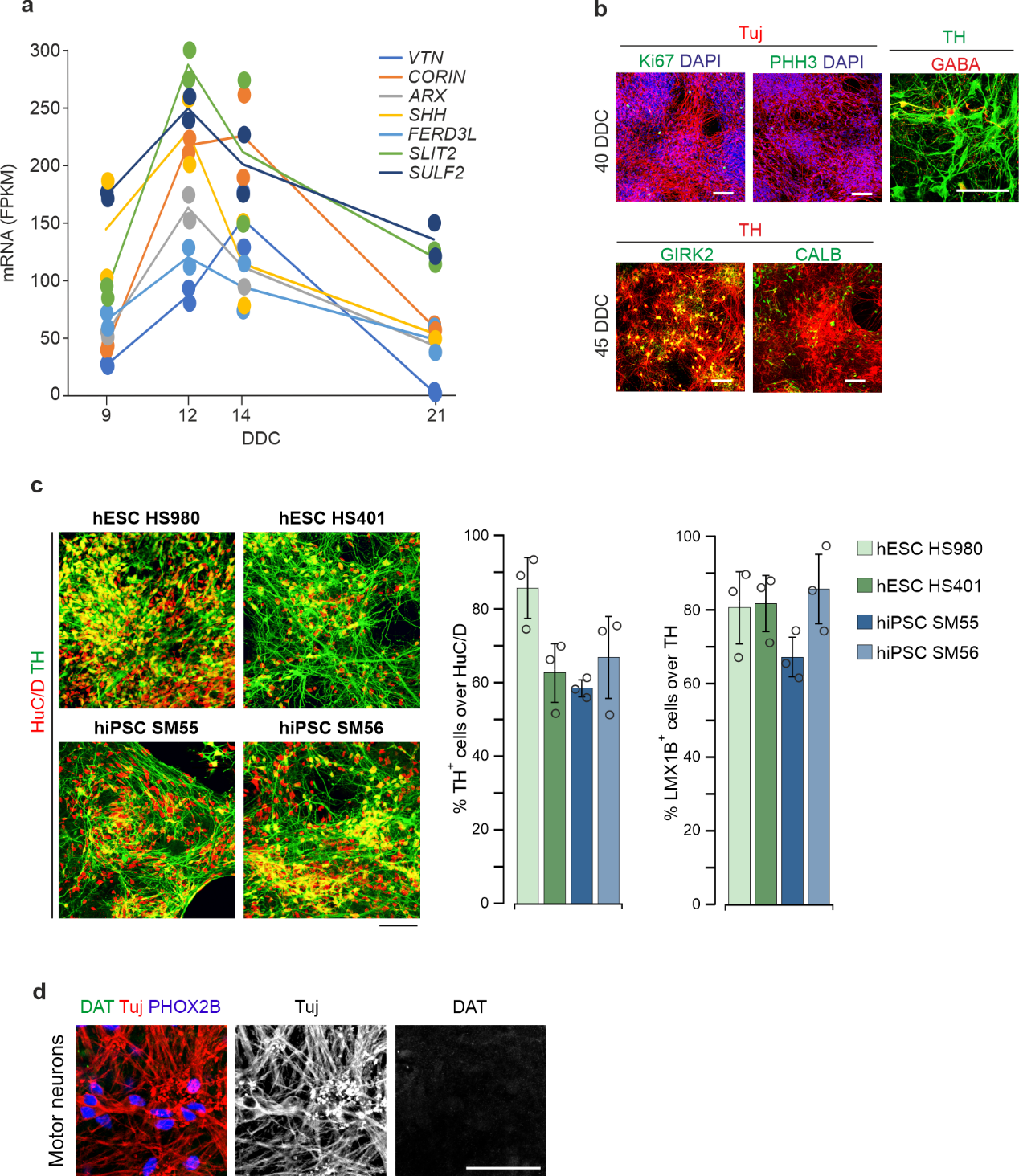
**

**Supplementary Fig. 5: Characterization of neurogenesis and differentiation in cultures differentiated in dSMADi+RA^2D^+SAG condition. a** RNA-seq data showing expression levels of floor plate genes between 9-21 DDC in cultures differentiated in dSMADi+RA^2D^+SAG in two independent experiments. **b** Immunocytochemistry of Tuj together with Ki67 or PPH3 and TH with GABBA in 40 DDC cultures, and of TH with GIRK2 or CALB1 in 45 DDC cultures. **c** Immunocytochemistry of TH and the neuronal marker HuC/D (left panel) and quantifications of the number of neurons expressing TH and of TH^+^ cells expressing LMX1B (right panel) in cultures at 40 DDC differentiated in dSMADi+RA^2D^+SAG condition for all four hPSC-lines examined in this study (values, mean±SD, n=3). **d** Validation of the specificity of DAT immunocytochemistry using a motor neuron differentiation culture as a negative control. Scale bars, 100 µm in panels **b,c**; 50 µm in panel **d**.

**
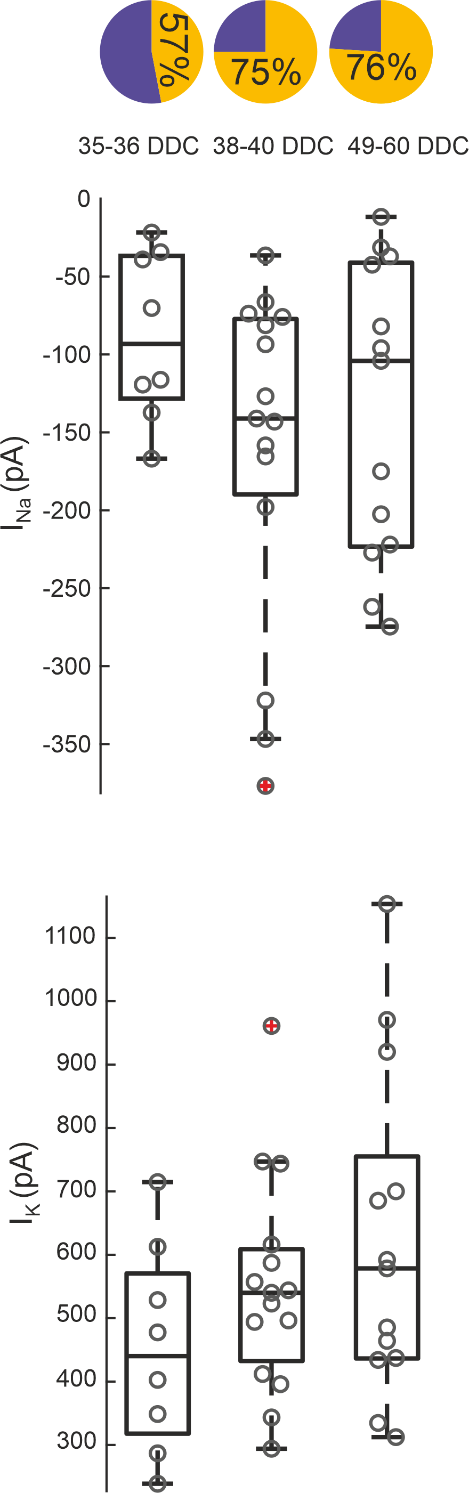
**

**Supplementary Fig. 6: Characterization of neuronal properties in cultures differentiated in dSMADi+RA^2D^+SAG condition.** Na^+^ and K^+^ currents of cells that showed a Na^+^ current. Pie charts denote the percentage of patched cells that show a Na^+^ current (yellow) and the percentage of cells that are inactive (purple) (n = 54 cells in total, 35-36 DDC: 8/17 cells, 38-40 DDC: 15/20 cells, 49-60 DDC: 13/17 cells). Boxplots: the band denotes the median, the box denotes 25th to 75th percentile, and the whiskers cover the extend of all data points, red point corresponds to an outlier (n = 36/54 cells (36 out of 54 patched cells show sodium current)).

**
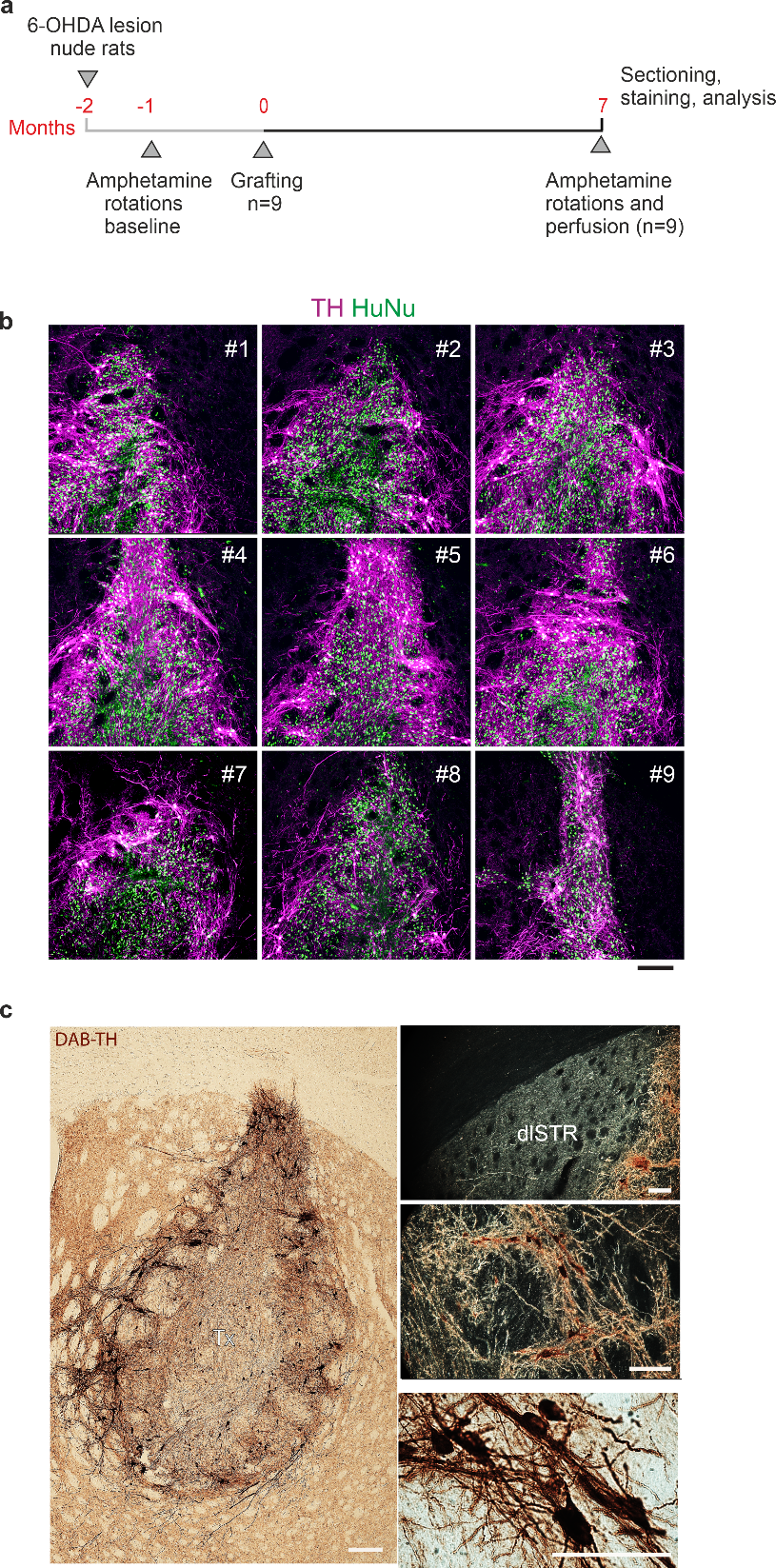
**

**Supplementary Fig. 7: Timeline and outcome of hESC-derived NPC grafting in rat model of PD. a** Schematic of the timeline of transplantation assay and analysis in 6-OHDA lesioned rats. **b** Immunohistological analysis of the expression of the human marker (HuNu) and the dopaminergic neuron marker (TH) in grafts of all transplanted rats (n=9). **c** DAB staining of graft-derived TH^+^ neurons innervating the surrounding dorsolateral striatum (dlSTR). Scale bars, 200µm (**b,c** left panel); 100 µm (**c** right panel).

| Gene | Log10.FC | p-value (t-test) | FDR (t-test) |
| --- | --- | --- | --- |
| *NANOG* | -7.408 | 0.004 | 0.26 |
| *GDF3* | -7.012 | 0.0029 | 0.26 |
| *LEFTY1* | -5.139 | 0.26 | 0.64 |
| *NODAL* | -4.52 | 0.32 | 0.67 |
| *LEFTY2* | -2.658 | 0.0011 | 0.26 |
| *TIMP4* | -1.79 | 0.059 | 0.37 |
| *TDGF1* | -1.693 | 0.054 | 0.36 |
| *IDO1* | -1.237 | 0.00057 | 0.26 |
| *PRDM14* | -1.093 | 0.0028 | 0.26 |
| *CD9* | -0.958 | 0.00022 | 0.26 |
| *POU5F1* | -0.897 | 0.014 | 0.27 |
| *FOXO1* | -0.765 | 0.092 | 0.43 |
| *ZFP42* | -0.655 | 0.023 | 0.3 |
| *DNMT3B* | -0.652 | 0.011 | 0.27 |
| *ETV5* | -0.414 | 0.22 | 0.6 |
| *ETV1* | -0.351 | 0.17 | 0.54 |
| *ETV4* | -0.334 | 0.024 | 0.3 |
| *KLF7* | -0.189 | 0.11 | 0.46 |
| *CDH1* | -0.156 | 0.094 | 0.43 |
| *SOX3* | 0.246 | 0.16 | 0.53 |
| *RARA* | 0.317 | 0.07 | 0.39 |
| *DLK1* | 0.666 | 0.0092 | 0.26 |
| *HES1* | 0.721 | 0.006 | 0.26 |
| *NCAM1* | 0.782 | 0.066 | 0.38 |
| *NRG1* | 0.855 | 0.044 | 0.34 |
| *POU3F1* | 0.857 | 0.018 | 0.29 |
| *SOX21* | 0.976 | 0.043 | 0.34 |
| *NRCAM* | 1.035 | 0.054 | 0.36 |
| *FOXJ1* | 1.12 | 0.052 | 0.36 |
| *CYP26A1* | 1.294 | 0.089 | 0.42 |
| *NR2F2* | 1.495 | 0.012 | 0.27 |
| *SULF1* | 1.566 | 0.011 | 0.27 |
| *PAX6* | 1.784 | 0.049 | 0.35 |
| *RFX4* | 2.005 | 0.085 | 0.42 |
| *SOX1* | 2.175 | 0.046 | 0.34 |
| *ZEB2* | 2.306 | 0.093 | 0.43 |
| *RARB* | 2.419 | 0.0046 | 0.26 |
| *HOXA1* | 3.662 | 0.026 | 0.3 |
| *GBX2* | 4.301 | 0.33 | 0.67 |
| *ZBTB16* | 6.038 | 0.0081 | 0.26 |

**Supplementary Table 1.** RNA-seq fold change in expression of genes associated with pluripotency or neuroectodermal fate in dSMADi+RA2D cultures at 2 DDC relative to ESCs. For log transformation, values FPKM=0 were replaced with 10^-6^. Statistical significance of differential expression was estimated using paired Student’s t test, assuming unequal variance and applying the Welch degrees of freedom modification with R function t-test. The p-values were adjusted for multiple testing by calculating the false discovery rate (FDR) by Benjamini and Hochberg’s method.

| **Gene** | **log2.FC** | **p-value (t-test)** | **FDR (t-test)** |
| --- | --- | --- | --- |
| *VTN* | -5.414 | 0.0088 | 0.094 |
| *NTN1* | -1.943 | 0.26 | 0.62 |
| *CORIN* | -1.891 | 0.015 | 0.13 |
| *ARX* | -1.335 | 0.044 | 0.23 |
| *SHH* | -1.005 | 0.18 | 0.51 |
| *FERD3L* | -0.944 | 0.18 | 0.51 |
| *SLIT2* | -0.75 | 0.23 | 0.59 |
| *SULF2* | -0.561 | 0.15 | 0.46 |
| *ALCAM* | -0.326 | 0.64 | 0.78 |
| *TUBB3* | 2.236 | 0.076 | 0.32 |
| *DCX* | 4.2 | 0.01 | 0.1 |
| *NR4A2* | 5.009 | 0.00034 | 0.015 |
| *STMN2* | 5.112 | 0.011 | 0.11 |
| *ASCL1* | 5.732 | 0.0036 | 0.057 |
| *NEUROD4* | 6.36 | 0.000035 | 0.0053 |
| *NEUROG2* | 9.563 | 0.0021 | 0.042 |
| *NEUROG1* | 15.631 | 0.15 | 0.47 |
| *TH* | 22.834 | 0.000015 | 0.0033 |

**Supplementary Table 2.** RNA-seq fold change in expression of genes associated with floorplate or neurogenesis in dSMADi+RA2D cultures at 21DDC relative to 14DDC. For log transformation, values FPKM=0 were replaced with 10^-6^. Statistical significance of differential expression was estimated using paired Student’s t test, assuming unequal variance and applying the Welch degrees of freedom modification with R function t-test. The p-values were adjusted for multiple testing by calculating the false discovery rate (FDR) by Benjamini and Hochberg’s method.

| **Species** | **Gene** | **Full gene name** | **Primer sequence (Fwd 5’-3’/Rev 5’-3’)** |
| --- | --- | --- | --- |
| Hs | *CORIN* | Corin, serin peptidase | CATATCTCCATCGCCTCAGTTG GGCAGGAGTCCATGACTGT |
| Hs | *CYP26A1* | Cytochrome P450 Family 26 Subfamily A member 1 | AGGAAATGACCCGCAATCTC  GAATGTTCTGCTCGATGCG |
| Hs | *EN1* | Engrailed 1 | CGTGGCTTACTCCCCATTTA  TCTCGCTGTCTCTCCCTCTC |
| Hs | *FOXA2* | Forkhead box A2 | CCGTTCTCCATCAACAACCT GGGGTAGTGCATCACCTGTT |
| Hs | *FOXG1* | Forkhead box G1 | TGGCCCATGTCGCCCTTCCT GCCGACGTGGTGCCGTTGTA |
| Hs | *GAPDH* | Glyceraldehyde-3-Phosphate Dehydrogenase | Prime Time qPCR primers Pre-designed IDT : exon: 2-3 |
| Hs | *HOXA2* | Homeobox A2 | ACAGCGAAGGGAAATGTAAAAGC GGGCCCCAGAGACGCTAA |
| Hs | *HOXA3* | Homeobox A3 | GTCAAACCCCTGTCAGAGTG  GCATTATAAGCGAACCCGTTG |
| Hs | *HOXB2* | Homeobox B2 | TTTCACCAGTACGCTCTGTG  TTTTCCAGTAGACGGCCAAG |
| Hs | *HOXB4* | Homeobox B4 | CTGGATGCGCAAAGTTCAC/TTCCTTCTCCAGCTCCAAGA |
| Hs | *LMX1A* | LIM homeobox transcription factor a | Prime Time qPCR primers Pre-designed IDT: exon: 3-4 |
| Hs | *LMX1B* | LIM homeobox transcription factor b | CTTAACCAGCCTCAGCGACT  TCAGGAGGCGAAGTAGGAAC |
| Hs | *NKX2.1* | NK2 homeobox 1 | AGGGCGGGGCACAGATTGGA GCTGGCAGAGTGTGCCCAGA |
| Hs | *NKX2.2* | NK2 homeobox 2 | Prime Time qPCR primers Pre-designed IDT |
| Hs | *NKX6.1* | NK6 homeobox 1 | Prime Time qPCR primers Pre-designed IDT : exon: 2-3 |
| Hs | *OTX1* | Orthodenticle homeobox 1 | TATAAGGACCAAGCCTCATGGC TTCTCCTCTTTCATTCCTGGGC |
| Hs | *OTX2* | Orthodenticle homeobox 2 | ACAAGTGGCCAATTCACTCC GAGGTGGACAAGGGATCTGA |
| Hs | *PAX6* | Paired box 6 | ACCACACCGGTTTCCTCCTTCACA TTGCCATGGTGAAGCTGGGCAT |
| Hs | *PHOX2A* | Paired Like Homeobox 2A | Prime Time qPCR primers Pre-designed IDT : exon: 2-3 |
| Hs | *PHOX2B* | Paired Like Homeobox 2B | Prime Time qPCR primers Pre-designed IDT : exon: 2-3 |
| Hs | *SIX3* | SIX homeobox 3 | ACCGGCCTCACTCCCACACA CGCTCGGTCCAATGGCCTGG |
| Hs | *TH* | Tyrosine hidroxylase | CGGGCTTCTCGGACCAGGTGTA CTCCTCGGCGGTGTACTCCACA |
| Hs | *VTN* | Vitronectin | Prime Time qPCR primers Pre-designed IDT : exon: 4-5 |
| Hs | *SLC18A2* | Solute carrier family 18 member A2 | Prime Time qPCR primers Pre-designed IDT : exon: 2-3 |
| Hs | *SLC6A3* | Solute carrier family 6 member A3 | Prime Time qPCR primers Pre-designed IDT : exon: 3-4 |
| Hs | *GIRK2*  *(KCNJ6)* | Potassium Inwardly Rectifying Channel Subfamily J | GATGGGAAACTGTGCCTGAT CTCCGAGGTCTGTTTGGATTT |
| Hs | *SOX6* | SRY-box transcription factor 6 | Prime Time qPCR primers Pre-designed IDT : exon: 17-18 |
| Hs | *ALDH1A1* | Aldehyde dehydrogenase 1 family member A1 | Prime Time qPCR primers Pre-designed IDT : exon: 12-13 |

**Supplementary Table 3.** Primers used for qPCR analysis.

| **Antibodies** | **Supplier** | **Catalog No** | **Dilution** |
| --- | --- | --- | --- |
| Rabbit polyclonal anti-OCT4 | Santa Cruz Biotechnology | sc-9081 | 1:1000 (IHC) |
| Rabbit polyclonal anti-OCT4 | Cell Signaling | 2750 | 1:2000 (IHC)  1:4000 (WB) |
| Goat polyclonal anti-SOX1 | R&D Systems | AF-3369 | 1:2000 (IHC)  1:4000 (WB) |
| Mouse monoclonal anti-ACTIN | Seven Hills Bioreagents | LMAB-C4 | 1:4000 (WB) |
| Rabbit polyclonal anti-GAPDH | Invitrogen | PA1-987 | 1:2000 (WB) |
| Rabbit polyclonal anti-PAX6 | Sigma-Aldrich | HPA030775 | 1:1000 (IHC) |
| Goat polyclonal anti-OTX2 | R&D Systems | AF-1979 | 1:2000 (IHC) |
| Rabbit polyclonal anti-FOXG1 | Abcam | ab18259 | 1:2000 (IHC) |
| Rabbit polyclonal anti-HOXA2 | Sigma-Aldrich | HPA029774 | 1:1000 (IHC) |
| Mouse monoclonal anti-HOXB4 | DSHB | I12 | 1:20 (IHC) |
| Guinea-pig polyclonal anti-LMX1B | home made | N/A | 1:6000 (IHC) |
| Mouse monoclonal anti-NKX2.1 | Abcam | ab220211 | 1:1000 (IHC) |
| Mouse monoclonal anti-NKX2.2 | DSHB | 74.5A5 | 1:50 (IHC)  1:100 (WB) |
| Rabbit polyclonal anti-LMX1A | Merck Millipore | AB10533 | 1:3000 (ICH) 1:4000 WB |
| Guinea-pig polyclonal anti-PHOX2B | home made | N/A | 1:12000 (IHC) |
| Goat polyclonal anti-FOXA2 | R&D Systems | AF-2400 | 1:1000(ICH)  1:4000 (WB) |
| Rabbit polyclonal anti-NURR1 | Santa Cruz Biotechnology | sc-991 | 1:300 (IHC) |
| Rabbit polyclonal anti-BARHL1 | Novus Biologicals | NBP1-86513 | 1:500 (IHC) |
| Sheep plyclonal anti-PITX2 | R&D Systems | AF7388 | 1:500 (IHC) |
| Mouse monoclonal anti-NKX6.1 | DSHB | F65A2 | 1:100 (IHC) |
| Mouse monoclonal anti-β-CATENIN | Santa Cruz Biotechnology | sc-7963 | 1:1000 (IHC) |
| Mouse monoclonal anti-PHOX2A | Santa Cruz Biotechnology | sc-81978 | 1:1000 (IHC) |
| Mouse monoclonal anti-EN1 | DSHB | 4G11 | 1:20 (IHC) |
| Rabbit polyclonal anti-GIRK2 | Alamone Labs | APC006 | 1:500 (IHC) |
| Mouse monoclonal anti-Tuj1 | Sigma-Aldrich | T8578 | 1:2000 (IHC) |
| Rabbit polyclonal anti-TH | Novus Biologicals | NB300-109 | 1:1000 (IHC) |
| Sheep polyclonal anti-TH | Novus Biologicals | NB300-110 | 1:1000 (IHC) |
| Mouse monoclonal anti-TH | Sigma-Aldrich | T2928 | 1:1000 (IHC) |
| Rabbit polyclonal anti-5-HT | Immunostar | 20080 | 1:5000 (IHC) |
| Goat polyclonal anti-5-HT | Immunostar | 20079 | 1:1000 (IHC) |
| Rabbit polyclonal anti-CALBINDIN | Sigma-Aldrich | HPA023099 | 1:1000 (IHC) |
| Mouse monoclonal anti-MAP2 | R&D Systems | MAB8304 | 1:2000 (IHC) |
| Rabbit polyclonal anti-DAT | Merck Millipore | AB1766 | 1:500 (IHC) |
| Rabbit polyclonal anti-GABA | Sigma-Aldrich | A2052 | 1:1500 (IHC) |
| Rabbit polyclonal anti-SYNAPTOPHYSIN | Zymed | 18-0130 | 1:200(IHC) |
| Guinea-pig polyclonal anti-PITX3 | Johan Ericson/Thomas Perlmann, KI | N/A | 1:20000 (IHC) |
| Mouse monoclonal anti-SHH | DSHB | 5E1 | 1:20 (IHC) |
| Mouse monoclonal anti-PAX3 | DSHB | PAX3 clone C2 | 1:40 (WB) |
| Rat monoclonal anti-Ki67 | Invitrogen | 14-5698-82 | 1:1000 (IHC) |
| Rabbit anti-LMX1A | Dr. M. German, San Francisco, CA | N/A | 1:6000 (IHC) |
| Mouse monoclonal anti-ISL1 | DSHB | 40.3A4 | 1:100 (IHC) |
| Rabbit polyclonal anti-PRPH | Merk Millipore | AB1530 | 1:1000 (IHC) |
| Rabbit polyclonal anti-5HTR-1A | Santa Cruz Biotechnology | sc-10801 | 1:200 (IHC) |
| Mouse monoclonal anti-GATA3 | Santa Cruz Biotechnology | sc-268 | 1:300 (IHC) |
| Mouse monoclonal anti- HuC/D | Molecular probes | A21271 | 1:1000 (IHC) |
| Rabbit polyclonal anti-SERT | Alomone labs | AMT-004 | 1:500 (IHC) |
| Goat polyclonal anti-TPH2 | Everest Biotech | EB07050 | 1:500 (IHC) |
| Mouse monoclonal anti-hNCAM | Santa Cruz Biotechnology | sc-106 | 1:500 (IHC) |
| Mouse monoclonal anti-HuNu | Chemicon | MAB1281 | 1:1000 (IHC) |
| Rat anti-CORIN | R&D Systems | MAB 2209 | 1:500 (IHC) |
| Goat polyclonal anti-Neurogenin2 | Santa Cruz Biotechnology | sc-19233 | 1:200 (IHC) |
| Mouse anti-NESTIN | Merk Millipore | MAB5326 | 1:1000 (IHC) |
| Alexa Fluor 488 donkey anti-rabbit IgG | Invitrogen | A21206 | 1:500 |
| Alexa Fluor 555 donkey anti-rabbit IgG | Invitrogen | A31572 | 1:500 |
| Alexa Fluor 647 donkey anti-rabbit IgG | Invitrogen | A31573 | 1:500 |
| Alexa Fluor 488 donkey anti-mouse IgG | Invitrogen | A21202 | 1:500 |
| Alexa Fluor 555 donkey anti-mouse IgG | Invitrogen | A31570 | 1:500 |
| Alexa Fluor 647 donkey anti-mouse IgG | Invitrogen | A31573 | 1:500 |
| Alexa Fluor 647 goat anti-mouse IgG2b | Invitrogen | A21242 | 1:500 |
| Alexa Fluor 488 goat anti-mouse IgG1 | Invitrogen | A21121 | 1:500 |
| Alexa Fluor 555 goat anti-mouse IgG1 | Invitrogen | A21127 | 1:500 |
| Alexa Fluor 647 goat anti-mouse IgG1 | Invitrogen | A21240 | 1:500 |
| Alexa Fluor 488 donkey anti-goat IgG | Invitrogen | A11055 | 1:500 |
| Alexa Fluor 555 donkey anti-goat IgG | Invitrogen | A21432 | 1:500 |
| Alexa Fluor 647 donkey anti-goat IgG | Invitrogen | A21447 | 1:500 |
| Alexa Fluor 488 donkey anti-guinea pig IgG | Invitrogen | A11073 | 1:500 |
| Alexa Fluor 555 donkey anti- guinea pig IgG | Invitrogen | A21435 | 1:500 |
| Alexa Fluor 647 donkey anti- guinea pig IgG | Invitrogen | A21450 | 1:500 |
| Mouse anti-goat IgG, HRP conjugated | Invitrogen | 31400 | 1:5000 |
| goat anti-rabbit IgG, HRP conjugated | Invitrogen | 31460 | 1:5000 |
| goat anti-mouse IgG, HRP conjugated | Invitrogen | 31430 | 1:5000 |

**Supplementary Table 4**. List of primary and secondary antibodies.
