# Supplementary material for: Robust derivation of transplantable dopamine neurons from human pluripotent stem cells by timed retinoic acid delivery
Source: Nat Commun. 2022 Jun 1;13:3046. doi: 10.1038/s41467-022-30777-8 (PMC9160024; doi:10.1038/s41467-022-30777-8)
Supplement: Supplementary file 3 — Reporting Summary [file 41467_2022_30777_MOESM3_ESM.pdf]

nature portfolio

Corresponding author(s): Johan Ericson

Last updated by author(s): Mar 22, 2022

Reporting Summary

Nature Portfolio wishes to improve the reproducibility of the work that we publish. This form provides structure for consistency and transparency in reporting. For further information on Nature Portfolio policies, see our Editorial Policies and the Editorial Policy Checklist.

Statistics

For all statistical analyses, confirm that the following items are present in the figure legend, table legend, main text, or Methods section.

n/a	Confirmed	
☐	☒	The exact sample size (*n*) for each experimental group/condition, given as a discrete number and unit of measurement
☐	☒	A statement on whether measurements were taken from distinct samples or whether the same sample was measured repeatedly
☐	☒	The statistical test(s) used AND whether they are one- or two-sided<br>*Only common tests should be described solely by name; describe more complex techniques in the Methods section.*
☒	☐	A description of all covariates tested
☐	☒	A description of any assumptions or corrections, such as tests of normality and adjustment for multiple comparisons
☐	☒	A full description of the statistical parameters including central tendency (e.g. means) or other basic estimates (e.g. regression coefficient) AND variation (e.g. standard deviation) or associated estimates of uncertainty (e.g. confidence intervals)
☐	☒	For null hypothesis testing, the test statistic (e.g. *F*, *t*, *r*) with confidence intervals, effect sizes, degrees of freedom and *P* value noted<br>*Give P values as exact values whenever suitable.*
☒	☐	For Bayesian analysis, information on the choice of priors and Markov chain Monte Carlo settings
☒	☐	For hierarchical and complex designs, identification of the appropriate level for tests and full reporting of outcomes
☒	☐	Estimates of effect sizes (e.g. Cohen's *d*, Pearson's *r*), indicating how they were calculated

Our web collection on statistics for biologists contains articles on many of the points above.

Software and code

Policy information about availability of computer code

Data collection	Immunofluorescence images were acquired with a confocal Zeiss LSM 700 microscope with ZEN 2011 sofrware,<br>Zeiss AxioImager M2 fluorescent microscope with ZEN2 software , and Molecular Devices ImageXpress Micro, software version 6.0.<br>For neuron current recording, neurons were visualized using a DIC microscope (Scientifica, Uckfield, UK) with a 60x objective (Olympus, Tokyo, Japan). Signals were recorded with an Axon MultiCalmp 700B amplifier and digitized at 20 kHz with an Axon Digidata 1550B digitizer (Molecular Devices, San Jose, CA, USA).<br>Quantitative Real-Time PCR was performed in a 7500 Fast Real Time PCR system.<br>For RNA-seq experiments, RNA integrity was determined on an Agilent RNA 6000 Pico chip, using Agilent 2100 BioAnalyzer (Agilent Technologies). Samples were sequenced on NovaSeq6000 and HiSeq2500 instruments.<br>For western blot analysis, detection of HRP was performed on a ChemiDoc Imaging System (Bio-Rad).<br>For HLPC analysis, samples were analyzed in a HPLC system consisting of HTEC500 (Eicom, Kyoto, Japan) and a CMA/200 Refrigerated Microsampler (CMA Microdialysis, Stockholm, Sweden). Separation was achieved on a 200 x 2.0 mm Eicompak CAX column (Eicom). The chromatograms were recorded and integrated using the computerized data acquisition system Clarity (DataApex).
Data analysis	ImageXpress images were analyzed with MetaXpress Version 5.0. Confocal images were analyzed with ImageJ/Fiji 1.53, or CellProfiler 3.1.5 software. Average neurite length was quantified using NeuronJ plugin in ImageJ package (https://imagej.net/plugins/neuronj). The Bcl to FastQ conversion was performed using bcl2fastq_v2.19.1.403 from the CASAVA software suite. Reads were mapped to the human genome assembly, build GRCh37 (https://www.ncbi.nlm.nih.gov/assembly/GCF_000001405.13/) using STAR (v 2.6.1a). Gene level abundances were estimated as FPKMs using StringTie2. Statistical significance of differential expression was estimated with t-test, using p-values and false discovery rate (R package v. 3.6.2). Gene level abundances were estimated as FPKMs using StringTie2. Script to determine current amplitude in single cell recordings of dopamine neurons in cell culture have been deposited to Zenodo (https://zenodo.org/record/6367837#.YjSC3DUo9PY).

For manuscripts utilizing custom algorithms or software that are central to the research but not yet described in published literature, software must be made available to editors and reviewers. We strongly encourage code deposition in a community repository (e.g. GitHub). See the Nature Portfolio guidelines for submitting code & software for further information.

Data

Policy information about availability of data

All manuscripts must include a data availability statement. This statement should provide the following information, where applicable:

- Accession codes, unique identifiers, or web links for publicly available datasets
- A description of any restrictions on data availability
- For clinical datasets or third party data, please ensure that the statement adheres to our policy

Source Data for Figs. 1-6 and Supplementary Figs. 1-7 are provided with this paper.
RNA-seq datasets reported in this manuscript have been deposited to the Gene Expression Omnibus under accession number GSE147404 (https://www.ncbi.nlm.nih.gov/geo/query/acc.cgi?acc=GSE147404). Human genome assembly, build GRCh37 (https://www.ncbi.nlm.nih.gov/assembly/GCF_000001405.13/).

Field-specific reporting

Please select the one below that is the best fit for your research. If you are not sure, read the appropriate sections before making your selection.

☒ Life sciences ☐ Behavioural & social sciences ☐ Ecological, evolutionary & environmental sciences

For a reference copy of the document with all sections, see nature.com/documents/nr-reporting-summary-flat.pdf

Life sciences study design

All studies must disclose on these points even when the disclosure is negative.

Sample size	No statistical methods were used to predetermine sample size. The sample size was chosen based on previous publications. Please see: Dias et al., 2014 (PMID: 25467979), Dias et al.; 2020 (PMID: 32938678); Kirkeby et al., 2017 (PMID: 28094017)
Data exclusions	No data were excluded from analysis
Replication	Reproducibility was ensured by sampling from multiple biological replicates, i.e., from multiple rats or from multiple PSCs differentiations. The exact number of replicates in groups or independent biological experiments is mentioned in the figure legends .No results are included that were not observed in multiple experiments. All attempts at replication were successful.
Randomization	Cell differentiation experiments were not randomized but different members of the laboratory have performed independent cell differentiation experiments using this method. For microscopy image analysis, the regions and cells within the experimental group were randomly selected.<br>All animals used in transplantation experiments were female rats with approximately similar age, weight and genetic background.
Blinding	Investigators were not blind to subject groups because knowledge of experimental conditions was required during data collection and evaluation.

Reporting for specific materials, systems and methods

We require information from authors about some types of materials, experimental systems and methods used in many studies. Here, indicate whether each material, system or method listed is relevant to your study. If you are not sure if a list item applies to your research, read the appropriate section before selecting a response.

Materials & experimental systems

n/a	Involved in the study	
☐	☒	Antibodies
☐	☒	Eukaryotic cell lines
☒	☐	Palaeontology and archaeology
☐	☒	Animals and other organisms
☒	☐	Human research participants
☒	☐	Clinical data
☒	☐	Dual use research of concern

Methods

n/a	Involved in the study	
☒	☐	ChIP-seq
☒	☐	Flow cytometry
☒	☐	MRI-based neuroimaging

Antibodies

Antibodies used	Antibodies/ Supplier/ Catalog No/ Dilution

Rabbit polyclonal anti-OCT4/ Santa Cruz Biotechnology/ sc-9081/ 1:1000 (IHC)
Rabbit polyclonal anti-OCT4/ Cell Signaling/ 2750/ 1:2000 (IHC);1:4000 (WB)
Goat polyclonal anti-SOX1/ R&D Systems/ AF-3369/ 1:2000 (IHC);1:4000 (WB)
Mouse monoclonal anti-ACTIN/ Seven Hills Bioreagents/ LMAB-C4/ 1:4000 (WB)
Rabbit polyclonal anti-GAPDH/ Invitrogen/ PA1-987/ 1:2000 (WB)
Rabbit polyclonal anti-PAX6/ Sigma-Aldrich/ HPA030775/ 1:1000 (IHC)
Goat polyclonal anti-OTX2/ R&D Systems/ AF-1979/ 1:2000 (IHC)
Rabbit polyclonal anti-FOXG1/ Abcam/ ab18259/ 1:2000 (IHC)
Rabbit polyclonal anti-HOXA2/ Sigma-Aldrich/ HPA029774/ 1:1000 (IHC)
Mouse monoclonal anti-HOXB4/ DSHB/ I12/ 1:20 (IHC)
Guinea-pig polyclonal anti-LMX1B/ home made/ N/A/ 1:6000 (IHC)
Mouse monoclonal anti-NKX2.1/ Abcam/ ab220211/ 1:1000 (IHC)
Mouse monoclonal anti-NKX2.2/ DSHB/ 74.5A5/ 1:50 (IHC);1:100 (WB)
Rabbit polyclonal anti-LMX1A/ Merck Millipore/ AB10533/ 1:3000 (ICH);1:4000 WB
Guinea-pig polyclonal anti-PHOX2B/ home made/ N/A/ 1:12000 (IHC)
Goat polyclonal anti-FOXA2/ R&D Systems/ AF-2400/ 1:1000(ICH)
1:4000 (WB)
Rabbit polyclonal anti-NURR1/ Santa Cruz Biotechnology/ sc-991/ 1:300 (IHC)
Rabbit polyclonal anti-BARHL1/ Novus Biologicals / NBP1-86513/ 1:500 (IHC)
Sheep plyclonal anti-PITX2/ R&D Systems/ AF7388/ 1:500 (IHC)
Mouse monoclonal anti-NKX6.1/ DSHB/ F65A2/ 1:100 (IHC)
Mouse monoclonal anti-β-CATENIN/ Santa Cruz Biotechnology/ sc-7963/ 1:1000 (IHC)
Mouse monoclonal anti-PHOX2A/ Santa Cruz Biotechnology/ sc-81978/ 1:1000 (IHC)
Mouse monoclonal anti-EN1/ DSHB/ 4G11/ 1:20 (IHC)
Rabbit polyclonal anti-GIRK2/ Alamone Labs / APC006/ 1:500 (IHC)
Mouse monoclonal anti-Tuj1/ Sigma-Aldrich/ T8578/ 1:2000 (IHC)
Rabbit polyclonal anti-TH/ Novus Biologicals/ NB300-109/ 1:1000 (IHC)
Sheep polyclonal anti-TH/ Novus Biologicals/ NB300-110/ 1:1000 (IHC)
Mouse monoclonal anti-TH/ Sigma-Aldrich/ T2928/ 1:1000 (IHC)
Rabbit polyclonal anti-5-HT/ Immunostar/ 20080/ 1:5000 (IHC)
Goat polyclonal anti-5-HT/ Immunostar/ 20079/ 1:1000 (IHC)
Rabbit polyclonal anti-CALBINDIN/ Sigma-Aldrich/ HPA023099/ 1:1000 (IHC)
Mouse monoclonal anti-MAP2/ R&D Systems/ MAB8304/ 1:2000 (IHC)
Rabbit polyclonal anti-DAT/ Merck Millipore/ AB1766/ 1:500 (IHC)
Rabbit polyclonal anti-GABA/ Sigma-Aldrich/ A2052/ 1:1500 (IHC)
Rabbit polyclonal anti-SYNAPTOPHYSIN/ Zymed/ 18-0130/ 1:200(IHC)
Guinea-pig polyclonal anti-PITX3/ Johan Ericson/Thomas Perlmann, KI/ N/A/ 1:20000 (IHC)
Mouse monoclonal anti-SHH/ DSHB/ 5E1/ 1:20 (IHC)
Mouse monoclonal anti-PAX3/ DSHB/ PAX3 clone C2/ 1:40 (WB)
Rat monoclonal anti-Ki67/ Invitrogen/ 14-5698-82/ 1:1000 (IHC)
Rabbit anti-LMX1A/ Dr. M. German, San Francisco, CA/ N/A/ 1:6000 (IHC)
Mouse monoclonal anti-ISL1/ DSHB/ 40.3A4/ 1:100 (IHC)
Rabbit polyclonal anti-PRPH/ Merk Millipore/ AB1530/ 1:1000 (IHC)
Rabbit polyclonal anti-5HTR-1A/ Santa Cruz Biotechnology/ sc-10801/ 1:200 (IHC)
Mouse monoclonal anti-GATA3/ Santa Cruz Biotechnology/ sc-268/ 1:300 (IHC)
Mouse monoclonal anti- HuC/D/ Molecular probes/ A21271/ 1:1000 (IHC)
Rabbit polyclonal anti-SERT/ Alomone labs/ AMT-004/ 1:500 (IHC)
Goat polyclonal anti-TPH2/ Everest Biotech/ EB07050/ 1:500 (IHC)
Mouse monoclonal anti-hNCAM/ Santa Cruz Biotechnology/ sc-106/ 1:500 (IHC)
Mouse monoclonal anti-HuNu/ Chemicon/ MAB1281/ 1:1000 (IHC)
Rat anti-CORIN/ R&D Systems/ MAB 2209/ 1:500 (IHC)
Goat polyclonal anti-Neurogenin2/ Santa Cruz Biotechnology/ sc-19233/ 1:200 (IHC)
Mouse anti-NESTIN/ Merk Millipore/ MAB5326/ 1:1000 (IHC)
Alexa Fluor 488 donkey anti-rabbit IgG/ Invitrogen/ A21206/ 1:500
Alexa Fluor 555 donkey anti-rabbit IgG/ Invitrogen/ A31572/ 1:500
Alexa Fluor 647 donkey anti-rabbit IgG/ Invitrogen/ A31573/ 1:500
Alexa Fluor 488 donkey anti-mouse IgG/ Invitrogen/ A21202/ 1:500
Alexa Fluor 555 donkey anti-mouse IgG/ Invitrogen/ A31570/ 1:500
Alexa Fluor 647 donkey anti-mouse IgG/ Invitrogen/ A31573/ 1:500
Alexa Fluor 647 goat anti-mouse IgG2b/ Invitrogen/ A21242/ 1:500
Alexa Fluor 488 goat anti-mouse IgG1/ Invitrogen/ A21121/ 1:500
Alexa Fluor 555 goat anti-mouse IgG1/ Invitrogen/ A21127/ 1:500
Alexa Fluor 647 goat anti-mouse IgG1/ Invitrogen/ A21240/ 1:500
Alexa Fluor 488 donkey anti-goat IgG/ Invitrogen/ A11055/ 1:500
Alexa Fluor 555 donkey anti-goat IgG/ Invitrogen/ A21432/ 1:500
Alexa Fluor 647 donkey anti-goat IgG/ Invitrogen/ A21447/ 1:500
Alexa Fluor 488 donkey anti-guinea pig IgG/ Invitrogen/ A11073/ 1:500

Alexa Fluor 555 donkey anti- guinea pig IgG/ Invitrogen/ A21435/ 1:500
Alexa Fluor 647 donkey anti- guinea pig IgG/ Invitrogen/ A21450/ 1:500
Mouse anti-goat IgG, HRP conjugated/ Invitrogen/ 31400/ 1:5000
goat anti-rabbit IgG, HRP conjugated/ Invitrogen/ 31460/ 1:5000
goat anti-mouse IgG, HRP conjugated/ Invitrogen/ 31430/ 1:5000

Validation

Previously published antibodies or antibodies with company based validations were used for immunohistochemistry:

Rabbit anti-OCT4 (Santa Cruz Biotechnology, sc-9081): https://www.scbt.com/p/oct-3-4-antibody-h-134

Rabbit anti-OCT4 (Cell Signaling, 2750): https://www.cellsignal.com/products/primary-antibodies/oct-4-antibody/2750

Goat anti-SOX1 (R&D Systems, AF-3369): https://www.rndsystems.com/products/human-mouse-rat-sox1-antibody_af3369

Mouse anti-ACTIN (Seven Hills Bioreagents, LMAB-C4): https://www.sevenhillsbioreagents.com/products/anti-actin-mouse-c4

Rabbit anti-GAPDH (Invitrogen, PA1-987): https://www.thermofisher.com/antibody/product/GAPDH-Antibody-Polyclonal/PA1-987

Rabbit anti-PAX6 (Sigma-Aldrich, HPA030775): https://www.sigmaaldrich.com/SE/en/product/sigma/hpa030775

Goat anti-OTX2 (R&D Systems, AF-1979): https://www.rndsystems.com/search?keywords=AF-1979

Rabbit anti-FOXG1 (Abcam, ab18259): https://www.abcam.com/foxg1-antibody-ab18259.html

Rabbit anti-HOXA2 (Sigma-Aldrich, HPA029774): https://www.sigmaaldrich.com/SE/en/product/sigma/hpa029774

Mouse anti-HOXB4 (DSHB, I12): https://dshb.biology.uiowa.edu/I12-anti-Hoxb4

Guinea-pig anti-LMX1B (home made, N/A): Dias et al. Tgfβ Signaling Regulates Temporal Neurogenesis and Potency of Neural Stem Cells in the CNS. Neuron 84:927-939 (2014)

Mouse anti-NKX2.1 (Abcam, ab220211): https://www.abcam.com/ttf1-antibody-nx21690-ab220211.html

Mouse anti-NKX2.2 (DSHB, 74.5A5): Dias et al. Tgfβ Signaling Regulates Temporal Neurogenesis and Potency of Neural Stem Cells in the CNS. Neuron 84:927-939 (2014); https://dshb.biology.uiowa.edu/74-5A5

Rabbit anti-LMX1A (Merck Millipore, AB10533): https://www.sigmaaldrich.com/SE/en/product/mm/ab10533

Guinea-pig anti-PHOX2B (home made, N/A): Dias et al. Tgfβ Signaling Regulates Temporal Neurogenesis and Potency of Neural Stem Cells in the CNS. Neuron 84:927-939 (2014)

Goat anti-FOXA2 (R&D Systems, AF-2400): https://www.rndsystems.com/search?keywords=AF-2400

Rabbit anti-NURR1 (Santa Cruz Biotechnology, sc-991): https://www.scbt.com/p/nurr1-antibody-n-20

Rabbit anti-BARHL1 (Novus Biologicals, NBP1-86513): https://www.novusbio.com/products/barhl1-antibody_nbp1-86513

Sheep anti-PITX2 (R&D Systems, AF7388): https://www.rndsystems.com/products/human-pitx2-antibody_af7388

Mouse anti-NKX6.1 (DSHB, F65A2): https://dshb.biology.uiowa.edu/F65A2

Mouse anti-β-CATENIN (Santa Cruz Biotechnology, sc-7963): https://www.scbt.com/p/beta-catenin-antibody-e-5

Mouse anti-PHOX2A (Santa Cruz Biotechnology, sc-81978): https://www.scbt.com/p/phox2a-antibody-37k-2

Mouse anti-EN1 (DSHB, 4G11): https://dshb.biology.uiowa.edu/4G11

Rabbit anti-GIRK2 (Alamone Labs, APC006): https://www.alomone.com/p/anti-kir3-2-girk2/APC-006

Mouse anti-Tuj1 (Sigma-Aldrich, T8578): https://www.sigmaaldrich.com/SE/en/product/sigma/t8578

Rabbit anti-TH (Novus Biologicals, NB300-109): https://www.novusbio.com/products/tyrosine-hydroxylase-antibody_nb300-109

Sheep anti-TH (Novus Biologicals, NB300-110): https://www.novusbio.com/products/tyrosine-hydroxylase-antibody_nb300-110

Mouse anti-TH (Sigma-Aldrich, T2928): https://www.sigmaaldrich.com/SE/en/product/sigma/t2928

Rabbit anti-5-HT (Immunostar, 20080): https://www.immunostar.com/product/5-ht-serotonin-rabbit-antibody/

Goat anti-5-HT (Immunostar, 20079): https://www.immunostar.com/product/5-ht-serotonin-goat-antibody/

Rabbit anti-CALBINDIN (Sigma-Aldrich, HPA023099): https://www.sigmaaldrich.com/SE/en/product/sigma/hpa023099

Mouse anti-MAP2 (R&D Systems, MAB8304): https://www.rndsystems.com/products/human-map2-antibody-885232_mab8304

Rabbit anti-DAT (Merck Millipore, AB1766): https://www.sigmaaldrich.com/SE/en/product/mm/ab1766

Rabbit anti-GABA (Sigma-Aldrich, A2052): https://www.sigmaaldrich.com/SE/en/product/sigma/a2052

Rabbit anti-SYNAPTOPHYSIN (Zymed, 18-0130): Nieto-Estévez et al. Distinct Effects of BDNF and NT-3 on the Dendrites and Presynaptic Boutons of Developing Olfactory Bulb GABAergic Interneurons In Vitro. Cellular and Molecular Neurobiology (2021).

Guinea-pig anti-PITX3 (Johan Ericson/Thomas Perlmann, KI, N/A): Tiklová et al. Single-cell RNA sequencing reveals midbrain dopamine neuron diversity emerging during mouse brain development. Nat commun. 10:581 (2019)

Mouse anti-SHH (DSHB, 5E1): https://dshb.biology.uiowa.edu/5E1

Mouse anti-PAX3 (DSHB, PAX3 clone C2): https://dshb.biology.uiowa.edu/Pax3

Rat anti-Ki67 (Invitrogen, 14-5698-82): https://www.thermofisher.com/antibody/product/Ki-67-Antibody-clone-SolA15-Monoclonal/14-5698-82

Rabbit anti-LMX1A (Dr. M. German, San Francisco, CA, N/A): Marklund et al. Detailed expression analysis of regulatory genes in the early developing human neural tube. Stem Cells Dev 23: 5-15 (2014)

Mouse anti-ISL1 (DSHB, 40.3A4): Dias et al. Tgfβ Signaling Regulates Temporal Neurogenesis and Potency of Neural Stem Cells in the CNS. Neuron 84:927-939 (2014); https://dshb.biology.uiowa.edu/40-3A4

Rabbit anti-PRPH (Merk Millipore, AB1530): https://www.sigmaaldrich.com/SE/en/product/mm/ab1530

Rabbit anti-5HTR-1A (Santa Cruz Biotechnology, sc-10801): https://datasheets.scbt.com/sc-10801.pdf

Mouse anti-GATA3 (Santa Cruz Biotechnology, sc-268): Dias et al. Tgfβ Signaling Regulates Temporal Neurogenesis and Potency of Neural Stem Cells in the CNS. Neuron 84:927-939 (2014); https://www.scbt.com/p/gata-3-antibody-hg3-31

Mouse anti- HuC/D (Molecular probes, A21271): https://www.thermofisher.com/antibody/product/HuC-HuD-Antibody-clone-16A11-Monoclonal/A-21271

Rabbit anti-SERT (Alomone labs, AMT-004): https://www.alomone.com/p/anti-serotonin-transporter-sert-extracellular/AMT-004

Goat anti-TPH2 (Everest Biotech, EB07050): https://everestbiotech.com/product/goat-anti-tryptophan-hydroxylase-2-tph2-antibody/

Mouse anti-hNCAM (Santa Cruz Biotechnology, sc-106): https://www.scbt.com/p/ncam-antibody-eric-1

Mouse anti-HuNu (Chemicon, MAB1281): https://www.sigmaaldrich.com/SE/en/product/mm/mab1281

Rat anti-CORIN (R&D Systems, MAB 2209): https://www.rndsystems.com/search?keywords=MAB+2209

Goat anti-Neurogenin 2 (Santa Cruz, sc-19233): https://www.scbt.com/p/neurogenin-2-antibody-c-16

Mouse anti-NESTIN (Merk Millipore, MAB5326): https://www.sigmaaldrich.com/SE/en/product/mm/mab5326

Control differentiations were stained with secondary antibody alone.

Additionally, for western blots, samples that do not express the protein were used as controls.

Eukaryotic cell lines

Policy information about cell lines

Cell line source(s)	HS980 and HS401 ESC lines were provided by Drs Outi Hovatta and Fredrik Lanner, Karolinska Institutet .HS980 line was certified as GMP-compliant. (Main, H., Hedenskog, M., Acharya, G., Hovatta, O., & Lanner, F. (2020). Karolinska Institutet Human Embryonic Stem Cell Bank. Stem Cell Research, 45, 101810). Human iPSC lines SM55 and SM56 were provided by Drs Pamela J.McLean and Simon Moussaud (Neuroregeneration Lab within the Center for Regenerative Medicine, Mayo Clinic).

	Isolation of primary human skin fibroblasts (obtained with patient consent) and generation of iPSCs were approved by the Mayo Clinic. Institutional Review Board under IRB protocols (IRB 09-003803).
Authentication	Cell lines were authenticated by providers. The cells from all lines showed normal karyotype, morphology, and expressed markers of human pluripotent stem cells (defined by RNA sequencing, immunocytochemistry, western blot).
Mycoplasma contamination	All cell lines tested negative for mycoplasma contamination using LookOut Mycoplasma qPCR Detection Kit (Sigma)
Commonly misidentified lines (See ICLAC register)	No commonly misidentified cell lines were used

Animals and other organisms

Policy information about studies involving animals; ARRIVE guidelines recommended for reporting animal research

Laboratory animals	Adult female, athymic ''nude'' rats were purchased from Harlan/Envigo Laboratories (Hsd:RH-Foxn1rnu). Animals were at a minimum of 16 weeks at start of experiment (180 g).
Wild animals	This study did not involve wild animals
Field-collected samples	This study did not involve field-collected samples
Ethics oversight	All animal procedures were approved by the Malmö/Lund Ethics Committee for the use of laboratory animals (Malmö/Lunds djurförsöksetiska nämnd) and the Swedish Department of Agriculture (Jordbruksverket).

Note that full information on the approval of the study protocol must also be provided in the manuscript.
